# Initiation of human astrovirus type 1 infection was blocked by inhibitors of phosphoinositide 3-kinase

**DOI:** 10.1186/1743-422X-10-153

**Published:** 2013-05-16

**Authors:** Shoichiro Tange, Yan Zhou, Yuko Nagakui-Noguchi, Takeshi Imai, Akira Nakanishi

**Affiliations:** 1 Section of Gene Therapy, Department of Aging Intervention, National Center for Geriatrics and Gerontology, 35, Gengo, Morioka, Obu, Aichi 474-8522, Japan

**Keywords:** Astrovirus, Signal transduction, PI3K, ERK1/2

## Abstract

**Background:**

Upon initial contact with a virus, host cells activate a series of cellular signaling cascades that facilitate viral entry and viral propagation within the cell. Little is known about how the human astrovirus (HAstV) exploits signaling cascades to establish an infection in host cells. Recent studies showed that activation of extracellular signal-regulated kinase 1/2 (ERK1/2) is important for HAstV infection, though the involvement of other signaling cascades remains unclear.

**Methods:**

A panel of kinase blockers was used to search for cellular signaling pathways important for HAstV1 infection. To determine their impact on the infectious process, we examined viral gene expression, RNA replication, and viral RNA and capsid protein release from host cells.

**Results:**

Inhibitors of phosphoinositide 3-kinase (PI3K) activation interfered with the infection, independent of their effect on ERK 1/2 activation. Activation of the PI3K signaling cascade occurred at an early phase of the infection, judging from the timeframe of Akt phosphorylation. PI3K inhibition at early times, but not at later times, blocked viral gene expression. However, inhibiting the downstream targets of PI3K activation, Akt and Rac1, did not block infection. Inhibition of protein kinase A (PKA) activation was found to block a later phase of HAstV1 production.

**Conclusions:**

Our results reveal a previously unknown, essential role of PI3K in the life cycle of HAstV1. PI3K participates in the early stage of infection, possibly during the viral entry process. Our results also reveal the role of PKA in viral production.

## Background

The human astrovirus (HAstV), a member of the *Astroviridae* family, is a small (28–30 nm) non-enveloped virus with a 6.8-kb, positive-sense RNA genome bound at the 5′ end with the viral protein Vpg and polyadenylated at the 3′ end [1,2]. Human astroviruses cause gastroenteritis and are a leading cause of viral diarrhea in young children. HAstV type 1 (HAstV1) is the most prevalent of the eight known HAstV serotypes in patients with gastroenteritis. The viral genome of HAstV1 encodes two non-structural proteins, nsp1a and nsp1ab, and a structural protein, the viral capsid protein. The nsp1a protein is encoded by open reading frame (ORF) 1a, whereas the nsp1ab is produced by a translational frameshifting mechanism that begins by translating ORF1a, and then skips ORF1a’s stop codon by shifting to the overlapping ORF1b [3,4]. The nsp1a and nsp1ab polyproteins catalyze their own proteolytic processing to produce functional viral proteins, including Vpg and an RNA-dependent RNA polymerase [5]. These viral proteins are believed to concertedly modulate cellular function to facilitate viral propagation and directly participate in viral RNA replication [2]. The viral capsid protein, encoded by ORF2, is translated as an 87-kDa protein that undergoes maturational processing by cellular enzymes and trypsin to become the functional viral capsid [6,7]. The progeny virions produced in the host cell can be released without cell lysis, which appears to be linked to processing of the viral capsid protein by cellular caspases and may involve cellular apoptotic events [8-10].

Many viral infections are known to activate host cell signaling pathways. The initial contact of viruses with a host cell can trigger a series of signaling cascades that facilitate viral entry and viral propagation within the cell [11]. More specifically, this virus-induced signaling may activate cellular mechanisms that viruses rely on for initiating infection, such as endocytosis, macrocytosis, and phagocytosis as well as the mobilization of the actin cytoskeleton [12].

One important cellular signaling pathway is the phosphoinositide 3-kinase (PI3K)/Akt pathway, which regulates diverse cellular activities, including cell growth, proliferation, survival, apoptosis, metabolism, migration, and vesicular trafficking [13]. PI3K is activated when the Src homology domain of its regulatory subunit, p85, binds to auto-phosphorylated tyrosine kinase receptors, non-receptor tyrosine kinases, or some viral proteins in the cytoplasm [14]. The catalytic subunit of the activated PI3K, p110, then converts phosphatidylinositol 4,5-bisphosphate (PIP2) into the lipid messenger phosphatidylinositol (3,4,5)-trisphosphate (PIP3), which activates the downstream targets of PI3K. A primary target is Akt, a serine/threonine protein kinase that modulates diverse signaling pathways, such as cell survival, proliferation, migration, differentiation, and apoptosis [15]. The binding of PIP3 allows Akt to form a complex with PDK-1, which phosphorylates and activates Akt [15]. Another important target of PI3K is Rac1, a small G-protein involved in cytoskeletal remodeling during lamellipodium formation, cell-to-cell contact, and cell migration [16,17]. PIP3 activates Rac1 by mediating the activation of Rac1-specific guanine exchange factors, such as T-lymphoma invasion and metastasis actor 1 (Tiam1) or Vav1 [16,17].

Another important group of cellular signaling pathways are those of the mitogen-activated protein kinases (MAPKs), which include extracellular signal-regulated kinases 1 and 2 (ERK1/2), p38, and c-Jun N-terminal kinases (JNK). In the ERK1/2 pathway, signal is transduced by activated receptor tyrosine kinases, the small G protein Ras, Raf, and MAPK/ERK kinase1/2 (MEK1/2), which then activate ERK1/2 through phosphorylation. Activated ERK1/2 is known to regulate cell survival, proliferation, and differentiation [18].

The intracellular signaling events that control HAstV1 infection are still not well understood. A study by Moser and Schultz-Cherry [19] found that ERK1/2 are activated during the initial contact of HAstV with host cells and are important for establishing HAstV infection. In this study, we sought to identify additional signaling pathways that play important roles in HAstV1 infection. Our approach was to use a panel of kinase inhibitors to test whether the specific inhibition of individual signaling pathways interferes with HAstV1 infection. We found that inhibitors of PI3K activation blocked HAstV1 infection, despite the fact that ERK activation was not inhibited. This PI3K activation occurred at an early phase of the infection, and apparently did not involve PI3K-mediated phosphorylation of Akt. Thus, our results reveal a previously unknown role of PI3K in HAstV1 infection.

## Results

### Examining the effects of kinase inhibitors on viral capsid protein expression

**Figure 1:**
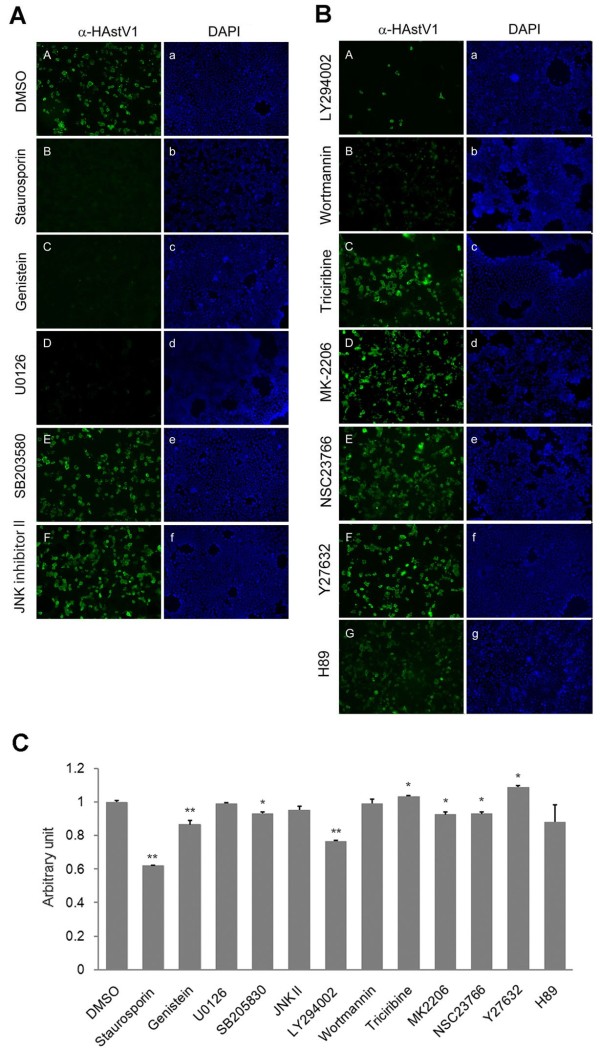
**Effects of kinase inhibitors on capsid protein expression following HAstV1 infection. (A)** Caco-2 cells were infected with HAstV1 (MOI = 0.22) in the presence of solvent alone **(**DMSO, panels **A** and **a)**, staurosporine **(**4 μM; **B** and **b)**, genistein **(**10 μM; **C** and **c)**, U0126 **(**20 μM; **D** and **d)**, SB 203580 **(**50 μM; **E** and **e)**, or JNK inhibitor II **(**50 μM; **F** and **f)**. The cells were fixed at 24 h post-infection (hpi), and then HAstV capsid protein was detected by immunofluorescence. Each pair of panels shows, for the same field of cells, the staining patterns for the viral capsid protein **(**anti-HAstV, **A** through **F)** and for cellular DNA (DAPI, a through f). **(B)** Caco-2 cells were infected with HAstV1 in the presence of various kinase inhibitors, LY294002 **(**50 μM; panels **A** and **a)**, wortmannin **(**1 μM; **B** and **b)**, Triciribine, **(**10 μM; **C** and **c)**, MK-2206 **(**10 μM; **D** and **d)**, NSC23766 **(**50 μM; **E** and **e)**, Y27632 **(**50 μM; **F** and **f)**, and H89 **(**10 μM; **G** and **g)**. Each pair of panels shows, for the same field of cells, the staining patterns for the viral capsid protein **(A** through **G)** and for cellular DNA **(a** through **g)**. **(C)** Viability of Caco-2 cells infected with HAstV1 in the presence of designated drugs was examined by colorimetric assay using WST-1 reagent (Takara). The absorbance of the cell culture medium was measured at 450 nm versus a 650-nm reference. The vertical axis indicates arbitrary unit divided by the mean value of a solvent-only (DMSO) control sample. The mean value obtained using three of each sample is presented as bar graph, with the standard deviation indicated as error bar. Statistical significance, compared with the solvent control (DMSO) is indicated (*P < 0.05; **P < 0.01).

To search for the signaling pathways that are important for HAstV1 infection, we examined various kinase blockers inhibitors (Table 1) for their ability to block HAstV1 infection of Caco-2 cells. Caco-2 cells were infected with HAstV1 in the presence or absence of each kinase inhibitor, and the presence of the inhibitor was maintained until 24 hours post-infection (hpi), when the cells were detected for viral capsid protein by immunofluorescence. While DMSO, the solvent for the inhibitors, did not interfere with viral gene expression (Figure 1A, panels A and a), 4 μM staurosporine (Figure 1A, B and b), a general kinase inhibitor, or 10 μM genistein (Figure 1A, C and c), a general inhibitor for tyrosine kinases, blocked viral gene expression. We noted that staurosporine treatment caused modest cellular toxicity, evident by nuclear staining with DAPI (Figure 1A, b) and by colorimetric assay for cell viability (Figure 1C). However, the almost complete absence of cells positive for viral antigen suggests that the drug was effective in blocking infection in the cells that survived drug treatment. Consistent with the previously reported requirement for ERK1/2 signaling in HAstV1 infection [19], U0126, a MEK1/2 inhibitor that blocks ERK1/2 phosphorylation, also blocked viral gene expression (Figure 1A, D and d). Other members of the MAPK family that we tested did not appear to be involved in establishing HAstV1 infection because neither 50 μM SB 203580, which blocks p38 activation (Figure 1A, E and e), nor 50 μM JNK inhibitor II, which selectively inhibits JNK (Figure 1A, F and f), had a significant effect on viral capsid gene expression.

**Table 1:** List of inhibitors used in this study

**Target molecules**	**Inhibitors**
General kinases	Staurosporine
Tyrosine kinases	Genistein
MEK1/2	U0126
p38α, p38β MAPK	SB203580
JNK 1/2/3	JNK inhibitor II
PI3K	LY294002
	Wortmannin
Akt	Triciribine
	MK2206
Rac1-specific GEF	NSC23766
ROCK	Y27632
PKA	H89

**Figure 2:**
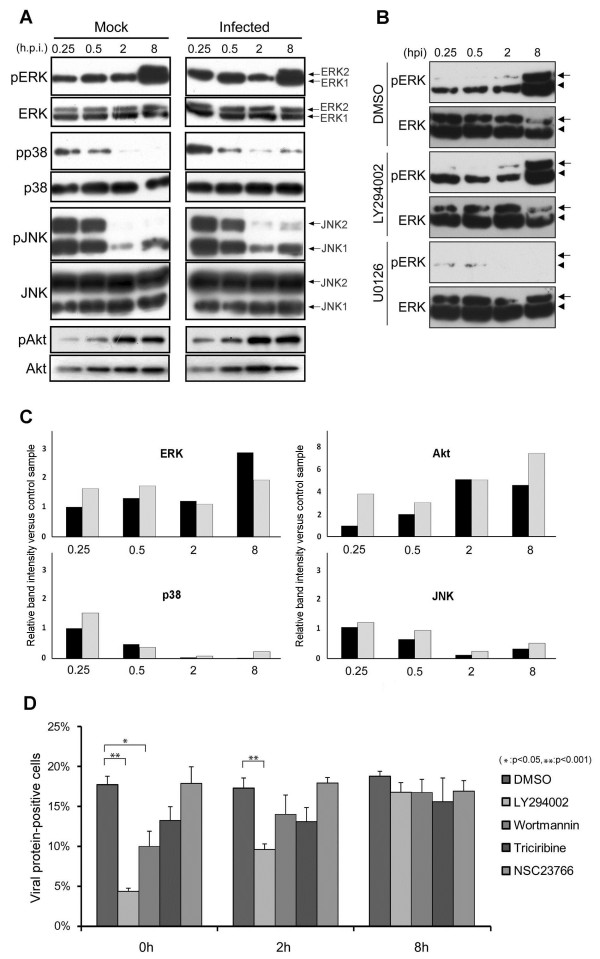
**Importance of timing of kinase inhibition during HAstV1 infection. (A)***Time course of activation of MAPKs and Akt*. Lysate was prepared from either mock-infected (Mock) or HAstV1-infected (Infected) Caco-2 cells at designated time points. The lysates were subjected to Western blot to detect either total kinases (ERK, p38, JNK, and Akt) or their phosphorylated forms [(pERK, pp38, pJNK, and pAkt) MAPKs and Akt]. The two bands in the “ERK” and “pERK” panels represent ERK1 (44 kDa) and ERK2 (42 kDa), respectively. The two bands in “JNK” and “pJNK” panels represent JNK1 (54 kDa) and JNK2 (46 kDa), respectively. **(B)***Effect of inhibitors on kinase activation.* Lysates prepared at designated time points from HAstV1-infected Caco-2 cells in the presence of solvent alone (DMSO), LY294002, or U0126 were subjected to Western blot for either total ERK (ERK) or its phosphorylated form (pERK). ERK1 and 2 bands are indicated by an arrowhead and an arrow, respectively. **(C)***Quantitative presentation of the Western blot signal shown in A*. Digital images of the bands were quantitated using ImageJ. The vertical line indicates the relative value of the signal intensity divided by the value of the band for each mock-infected sample, at 0.25 hpi, in each experimental group. The black and gray bars represent values for the mock- and HAstV1-infected sample, respectively. Note that, due to large differences, the scale of the vertical bar representing “Akt” differs from that of others. **(D)***Effects of inhibitors on capsid protein expression.* Caco-2 cells were infected and examined by immunofluorescence analysis as in Figure 1, except that solvent alone or inhibitors were added at indicated time points and maintained until fixing at 24 hpi. For each time point, the proportions of cells positive for capsid protein expression were examined statistically as described in Methods. (*P < 0.05; **P < 0.001).

We were also able to confirm that ERK1/2 activation occurs at an early stage of HAstV1 infection. The phosphorylation level of various kinases was examined at different times post-infection by Western blotting for both phosphorylated and phosphorylation-independent epitopes of each kinase (Figure 2A). The signal intensity of each band relative to that of each mock-infected sample at 0.25 hpi is presented in Figure 2C. Compared with that of the mock-infected sample, the phosphorylation levels of ERK1/2 were noticeably elevated at the early time points (0.25 hpi and 0.5 hpi) (Figure 2A, “pERK” and Figure 2C, “ERK”). Similarly, the p38 phosphorylation level appeared to be elevated at 0.25 hpi (Figure 2A, “pp38” and Figure 2C, “p38”). A marginal increase in the phosphorylation level of JNK was observed in the infected cells throughout the time points examined (Figure 2A, “pJNK” and Figure 2C, “JNK”). However, only the phosphorylation of ERK1/2, and not that of p38 and JNK, was necessary for infection, judged from the results of the capsid protein expression assay performed with inhibitors specific to these kinases (Figure 1A). We noted that the level of phosphorylated ERK1/2 increased at 8 hpi (Figure 2A, right “pERK”), an observation not reported earlier [19]. This is unlikely to be related to any infection event because phosphorylated ERK1/2 was similarly elevated at this time point in the mock-infected sample (left “pERK” panel).

Our search for additional HAstV1 infection-related signaling pathways uncovered evidence for the importance of PI3K activation. The PI3K inhibitor LY294002 (Figure 1B, panels A and a) effectively blocked post-infection viral capsid expression, whereas the other PI3K inhibitor, wortmannin (Figure 1B, panels B and b), was slightly less effective, evidenced by the unusual punctate signal of capsid protein. A possible explanation is that although more potent than LY294002 in inhibiting PI3K activation, wortmannin is only stable for a few minutes in the cellular environment [20], making the PI3K-inhibiting effect of LY294002 more apparent in a treatment that lasted 24 h.

One possibility consistent with the observed effect of PI3K inhibitors on HAstV1 infection is that they may have led to the inhibition of ERK phosphorylation. PI3K and MAP kinase pathways are known to crosstalk through small GTPases such as Ras and Raf1 [21,22]. To evaluate this possibility, the phosphorylation level of ERK in the presence or the absence of a PI3K blocker was analyzed by Western blotting (Figure 2B). We found that, unlike U0126, which abolished post-infection ERK phosphorylation (Figure 2B, “pERK” panel in the “U0126” group), LY294002 did not affect their phosphorylation (Figure 2B, “pERK” panel of the “LY294002” group). Thus, the PI3K inhibitor did not exert its effect through an interference with ERK activation, but acted on a distinct, essential process in HAstV1 infection.

We then asked whether known downstream targets of PI3K signaling, such as Akt, play a role in HAstV1 infection. Consistent with PI3K activation in the viral infection and with Akt being a target of activated PI3K, the extent of Akt phosphorylation was greater in the 0.25 h and 0.5 h post-infection samples than in the corresponding mock-infected control (Figure 2A, “pAkt” and Figure 2C, “Akt”). However, treatment with 10 μM triciribine (Figure 1B, C and c) or with 10 μM MK2206 (Figure 1B, D and d), both of which are known to inhibit Akt activation as well as Akt-mediated phosphorylation, had marginal effects on viral capsid expression. Examination of the phosphorylation level of Akt in the HAstV1-infected cells incubated with LY294002, wortmannin, triciribine, or MK2206 for 24 h showed that all but triciribine treatment effectively blocked the phosphorylation of Akt (Additional file 1 and see Discussion). In addition to the Akt-mediated cascade, Rac1 is also known to be targeted by PI3K activation [16]. Blocking Rac1 with 50 μM NSC23766, an inhibitor of Rac1-specific GEF, did not interfere with the infection (Figure 1B, E and e).

We also tested for the involvement of other signaling cascades. H89 blocks the activity of protein kinase A (PKA) by competing for the ATP binding site of PKA’s catalytic subunit. Y27632 inhibits Rho-associating protein kinase (ROCK). Neither inhibitor (at 10 μM and 50 μM, respectively) had an inhibitory effect on viral capsid protein expression (Figure 1B, G and g, and F and f), indicating that neither the PKA- nor the Rho-mediated pathway is significant for HAstV1 gene expression.

### Inhibitors that block Akt or Rac1 activation did not prevent the progression of infectious process

The increase in Akt activation at 0.25 and 0.5 h post-infection suggests that PI3K activation occurs at an early stage of infection. We also note that there is an increase of Akt phosphorylation at 8 hpi. To further examine if PI3K activation is needed in the initial phase of infection, inhibitors of PI3K, Akt, or Rac1 were added at 0, 2, or 8 hpi, and the proportion of cells positive for viral capsid expression was examined by immunofluorescence (Figure 2D). The Rac1 inhibitor NSC23766 did not block viral gene expression at any time point. The PI3K inhibitors LY294002 and wortmannin were effective in diminishing viral gene expression only when added at 0 or 2 hpi, at the time range of effectiveness similar to that of the ERK inhibitor [19]. Neither PI3K inhibitor was effective at 8 hpi. Although triciribine-treated cells appeared to exhibit a lower proportion of infected cells, the difference from the control sample was not significant. MK-2206, the other Akt inhibitor, did not affect viral gene expression (Figure 1B), suggesting that blockade of Akt had little effect on HAstV1 infection. Nonetheless, the results showing blockade of infection by PI3K inhibitors added at 0 and 2 hpi are consistent with the increased phosphorylation of Akt at 15 and 30 min post-infection seen in the Western blot (Figure 2A), which marks the increased PI3K kinase activity at those early time points, and suggest that PI3K activation is important at the initial stage of infection.

### Effects of kinase inhibitors on viral RNA replication

**Figure 3:**
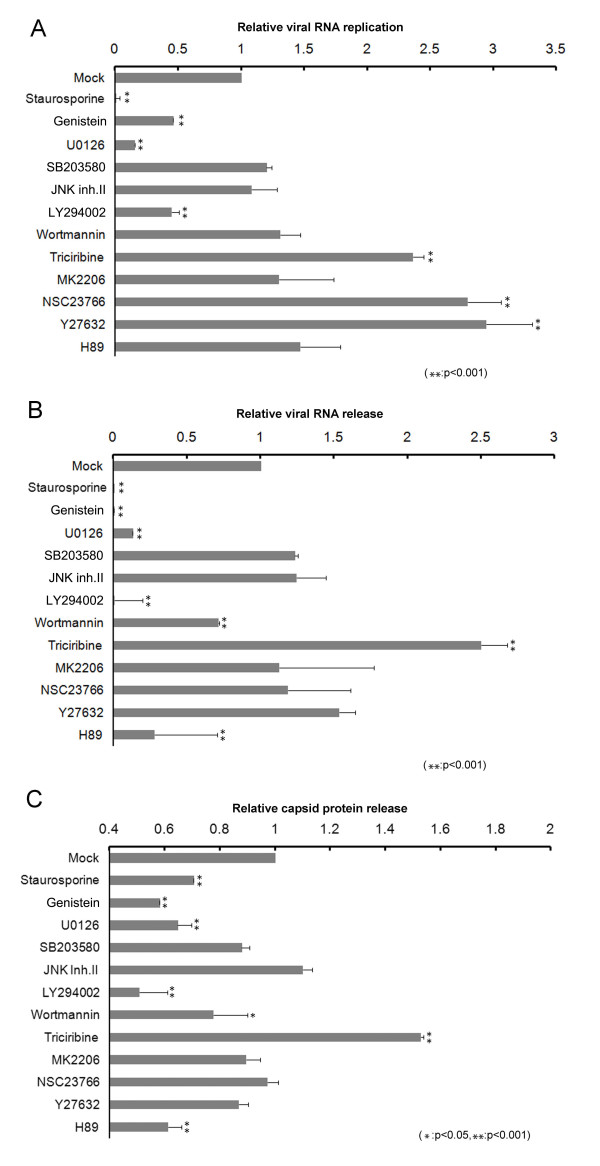
**Effects of kinase blockers on viral RNA replication and on the appearance of viral RNA and capsid protein in culture supernatant.** Caco-2 cells were infected with HAstV1 in the absence (Mock) or presence of various kinase inhibitors and examined for the following. **(A)***Effects on viral RNA replication.* Viral RNA replication in the infected cells was analyzed using quantitative real-time RT-PCR. For each drug treatment, the quantified amount of viral RNA, normalized to the amount of total RNA, was converted to a value relative to that of mock treatment. The mean relative value from four independent experiments, with the standard deviation indicated as error bar, is presented. **(B)***Effects on presence of viral RNA in culture supernatant.* The level of viral RNA in the post-infection culture supernatant was analyzed using real-time RT-PCR. For each drug treatment, the quantified amount of viral RNA was converted to a value relative to that of mock treatment. The mean relative value from three experiments, with the standard deviation indicated as error bar, is presented. **(C)***Effects on presence of viral capsid protein in culture supernatant.* The level of viral capsid antigen in the post-infection culture supernatant was measured using ELISA. The supernatant samples were prepared in triplicate, and the mean capsid antigen level for each drug treatment was further converted to a value relative to mock treatment. Statistically significant differences, determined as in Figure 2D, are indicated on the graphs.

The immunofluorescence detection of viral capsid protein offered a qualitative indication of whether a given kinase inhibitor affected the initiation of the infection processes leading to viral gene expression. In order to more quantitatively measure the effect of the drugs on viral propagation, the amount of viral RNA produced in the cells at 24 hpi in the presence or absence of the drugs was measured by quantitative real-time RT-PCR (Figure 3A). Cells treated with genistein, staurosporine, U0126, and LY294002 contained significantly lower amounts of viral RNA than cells treated with the solvent alone, consistent with the finding that these drugs were inhibitory to the expression of viral capsid. Although treatment with wortmannin could show inhibitory effect on viral capsid expression (Figure 1B), it did not translate into a significant effect on viral RNA replication (see Discussion). Not surprisingly, drugs that did not inhibit viral gene expression—inhibitors of MAPK p38s (SB203580), JNK (JNK inhibitor II), Akt (MK2206), and PKA (H89)—had no measurable effect on the extent of viral RNA replication. Treatment with triciribine, NSC23766, or Y27632 induced higher levels of RNA replication and did not inhibit the production of viral RNA. These results support the idea that PI3K activation is important for the initiation of viral infection via a non-Akt, non-Rac mediated pathway.

### Effects of kinase inhibitors on the release of viral RNA and capsid protein into cell culture supernatant

We next examined the effects of kinase inhibitors on the release of viral RNA, indicative of virion release, from the cell by measuring the level of viral RNA present in the culture supernatant of HAstV1-infected cells at 24 hpi (Figure 3B). In agreement with the result of our viral RNA replication analysis, treatment with staurosporine, genistein, U0126, or LY294002 greatly reduced the amount of viral RNA detected in the supernatant. Wortmannin treatment also lowered viral RNA content in the supernatant. Again, the Akt inhibitors triciribine and MK2206 exhibited a contrasting effect; triciribine apparently increased the amount of viral RNA in the culture supernatant as well as the extent of viral RNA replication (Figure 3A and B), whereas MK2206 had a marginal effect on viral RNA accumulation in both the cell and the culture supernatant. NSC23766 and Y27632, the inhibitors of Rac1 and ROCK, respectively, similarly failed to reduce either viral RNA replication or viral RNA release into the culture supernatant, consistent with their inability to prevent viral gene expression. However, the PKA inhibitor H89 showed some inhibitory effect on extracellular viral RNA accumulation, suggesting that PKA may play a role during virus release from the cell.

We tested the effects of kinase inhibitors on another marker for virus production and release, the presence of viral capsid in the culture supernatant of infected cells at 24 hpi (Figure 3C). The results are largely consistent with those of the analysis for viral RNA presence in the culture supernatant (Figure 3B). The same drugs that inhibited the viral capsid expression—genistein, staurosporine, U0126, and LY294002—also inhibited viral capsid accumulation in the culture supernatant. Wortmannin similarly lowered the level of extracellular capsid protein, consistent with its lowering of extracellular viral RNA (Figure 3B, Wortmannin). The contrasting effect of the Akt inhibitors triciribine and MK2206 seen in the assays for intracellular viral RNA production and extracellular viral RNA presence was also detected for the production of extracellular viral capsid. Again, the Rac1 and ROCK inhibitors NSC23766 and Y27632 had no effect, and the PKA inhibitor H89 showed some inhibitory effect on extracellular viral capsid production, in agreement with their respective effects on viral RNA (Figure 3B, NSC23766, Y27632, and H89).

## Discussion

In this study, a panel of kinase inhibitors was used to identify the cellular signal transduction pathways important for HAstV1 infection. We found that inhibitors of PI3K activation interfered with infection, independent of ERK activation. We showed that PI3K activation occurred at an early phase of infection and that the downstream targets Akt and Rac1 were not required for the infection. Blocking PI3K with either LY294002 or wortmannin diminished the production of viral particles, indicating that PI3K activation is important for HAstV1 infection. In addition, PKA was involved in some aspect of viral particle production. Taken together, our results reveal a previously unknown role of PI3K in establishing HAstV1 infection and PKA on viral production.

Our data indicate that very early in HAstV1 infection—within 30 min of the virions’ contact with the cells—the host Caco-2 cells activate signaling cascades that involve PI3K. Treating the cells with PI3K-specific inhibitors resulted in a block in HAstV1 infection that was detected at the levels of viral gene expression, viral RNA replication, and release of viral capsid and RNA from the cells. Although the phosphorylation of Akt did not appear to be essential for viral infection, the early time frame of PI3K activation indicated that PI3K was activated during an early phase of infection, perhaps at the step of viral entry. Similarly, ERK activation has been shown to be important early in HAstV1 infection [19]. Thus, both PI3K and ERK signaling appears to function during an early phase of HAstV1 infection, from viral cell entry to the initiation of viral gene expression.

During the course of this study, we also found that a PKA inhibitor decreased the release of viral components into the culture supernatant, but did not block capsid protein expression or viral RNA replication. A recent analysis of human cytomegalovirus infection using kinome profiling showed that PKA cascades are involved in the production of progeny virions by regulating the metabolic pathways of the host cells [23]. It would be interesting to examine whether PKA cascades metabolically control HAstV1 production.

Among the MAPK pathways, we found that both ERK and p38 were phosphorylated shortly after the HAstV1 virion makes contact with the cell, but only the activation of ERK appears to be essential for infection. Inhibiting ERK activation with U0126 blocked infection, but inhibiting p38 with SB 203580 did not. Similarly, Akt, one of the major downstream targets of PI3K, was found to be phosphorylated at Ser473 early in HAstV1 infection, though inhibitors of Akt, triciribine, and MK2206 did not seem to block viral capsid expression, viral RNA replication, or viral component release. Hence, the activation of p38 and Akt pathways upon infection appears to be either non-essential for HAstV1 infection or redundant with other pathways that could relay the essential signals for the infectious processes.

It is interesting to note that wortmannin treatment showed no blockade of RNA replication, but exhibited a block in viral release. Immunofluorescent detection of viral capsid protein revealed that treatment with wortmannin caused unusual punctate staining of the capsid protein, which suggests that the reagent failed to block viral entry, but was effective in delaying the process leading to capsid expression showing aberrant distribution. The time point examined for viral RNA replication, 24 hpi, may have been the point when viral RNA replication had already reached a plateau, but the inhibitory effect of wortmannin on the release of RNA and virion may have been visible because of the delay of the infectious process.

Treatment with triciribine enhanced viral RNA replication in HastV1-infected cells, which possibly caused the increased viral release that was inferred from the level of viral RNA and capsid protein in the culture supernatant (Figure 3). Surprisingly, we found that the Akt phosphorylation was not effectively blocked at 24 hpi (Additional file 1) and viral capsid release was enhanced in a dose-dependent manner (Additional file 2). We also noted that triciribine treatment slightly enhanced cell viability (Figure 1C). Overall, the treatment appeared to have a positive effect on viral propagation in our experiments, rather than an inhibitory effect. Similarly, treatment with NSC23766 or Y27632 increased the extent of viral RNA replication. Interestingly, a marked increase in the phosphorylated Akt level was observed in cells treated with each drug (Additional file 1). Akt activation is known to involve a feedback loop activating Rac1, led by ROCK inhibition using Y27632 [24]. Because Rho-family signaling events are known to involve balanced regulation [24], inhibition of another member of the Rho-family, Rac1, by NSC23766 could also have activated such a feedback loop. The activated Akt possibly caused an increase in protein synthesis, which could enhance viral RNA replication [14].

We noted that two Akt phosphorylation inhibitors affect HAstV1 infection differently. Triciribine apparently increased the amount of viral RNA and the release of viral RNA and capsid in the culture supernatant, whereas MK2206 did not (Figure 3). This difference could be due to a difference in the drugs’ inhibitory mechanisms. Triciribine inhibits Akt phosphorylation by binding to the PH domain of Akt, thereby blocking its recruitment to the plasma membrane [25], whereas MK2206 binds to the catalytic domain of Akt and inhibits its phosphorylation [26]. Triciribine is also known to inhibit cellular DNA synthesis [27]. Nonetheless, neither Akt inhibitor blocked viral infection.

In summary, our study has revealed that two signaling pathways, mediated by ERK and PI3K, are important for HAstV1 infection. The observation that specific, selective PI3K kinase inhibitors did not block ERK phosphorylation, yet exhibited inhibitory effect on infection, indicates that the PI3K-mediated cascade acts independent or downstream of that mediated by ERK (Figure 2B). The involvement of ERK activation is not uncommon in signaling during viral infection. ERK signaling has been shown to be important in the mobilization of receptors for the hepatitis C virus (HCV) [28]; in viral gene expression for respiratory syncytial virus [29], human cytomegalovirus [30], and Kaposi’s sarcoma-associated herpes virus (KSHV) [31]; in viral genome replication for the influenza virus [32] and mouse hepatitis virus [33]; in viral assembly for HCV [34]; and in viral release from host cells for Borna disease virus [35]. Similarly, PI3K-Akt activation is needed for viral entry for the influenza virus [36], avian leucosis retrovirus [37], and vaccinia virus [38], all of which are also functionally dependent on Akt activation, unlike the case with HAstV1 infection. An integration of multiple signaling cascades has been shown for KSHV infection, in which the FAK-Src-PI3K-PKC-MEK-ERK cascade is involved in viral early gene expression [39], and the PI3K-Akt-RhoA cascade, but not ERK activation, is important for viral entry [40]. An integration of the PI3K and ERK pathways was not observed in HAstV1 infection; rather, the signaling pathways appeared to be separate. Because such a pattern of kinase activation during infection has not been found for other viruses, our study has uncovered a unique signal transduction strategy of HAstV1 for establishing infection in host cells.

## Conclusions

A panel of kinase inhibitors was used to identify the cellular signal transduction pathways important for HAstV1 infection. Inhibitors that block PI3K activation were found to interfere with infection, independent of the process of ERK activation. PI3K activation occurred at an early phase of infection, and the downstream targets required for the infection were not Akt or Rac1. Moreover, PKA was found to be involved in some aspects of viral particle production. Our results reveal a previously unknown role of PI3K in establishing HAstV1 infection and PKA on viral production.

## Methods

### Virus and cells

The HAstV1 isolate was provided by Dr. Mitsuaki Oseto (Ehime Hygiene Environmental Institute, Japan). Caco-2 cells (from Dr. Naomi Sakon, Osaka Prefectural Institute of Public Health, originally given by Dr. Albert Z. Kapikian) were maintained in a culture medium (EMEM+) consisting of minimum essential medium with Eagle’s modification (EMEM) (catalog no. M4655; Sigma-Aldrich, St. Louis, MO, USA) supplemented with 1 mM sodium pyruvate, non-essential amino acids (Invitrogen, Green Island, NY, USA), and 10% fetal bovine serum.

### Preparation of virus stocks, quantitation of viral particles, and measurement of infectious titer

To prepare HAstV1 stocks, Caco-2 cells were infected with HAstV1 at approximately 100 viral particles per cell. The culture supernatant was collected 2 days after infection, freeze-thawed, cleared of cell debris by centrifugation, and stored in aliquots as HAstV1 stocks. These stocks typically contained about 10^9^ particles per mL.

The number of viral particles present in the viral preparations was determined from a measurement of RNA copy number obtained using real-time quantitative RT-PCR. Viral RNA was extracted from each sample of the viral preparations using the QIAamp Viral RNA Mini Kit (Qiagen, Hilden, Germany). The extracted RNA, along with a known amount of standard HAstV1 RNA (see below), was reverse-transcribed into cDNA using the Superscript III system (Invitrogen) with oligo-dT as the primer. For quantitating the copy number of the viral genome, cDNA was amplified using viral cDNA-specific primers, S3988-4008 (5′-GAGACATCTTTGGCATGTTGG-3′) and AS4193-4171 (5′-AGGAGCTTCCCATGGAGTGATTC-3′) with the Thunderbird q-PCR Kit (Toyobo Life Science, Osaka, Japan). Amplification proceeded through 40 cycles of denaturation at 94°C for 15 s, annealing at 62°C for 20 s, and extension at 72°C for 20 s in either a LightCycler 2.0 (Roche Applied Science, Penzberg, Germany) or a CFX-96 (Bio-Rad, Hercules, CA, USA). The cDNA copy number, derived from the fluorescence signals of the amplification products, was then converted into particle number.

Standard HAstV1 RNA was prepared by in vitro transcription using a T7 RiboMax Express Large Scale RNA Production System (Promega) and the template DNA pAVIC V (a gift of Dr. Albert Bosch, University of Barcelona), which harbors a molecular clone of HAstV1.

Infectious titer was determined using the method described by Mendez et al. [8]. In our study, infection with 100 particles per Caco-2 cell yielded approximately 20% of the cells positive for anti-HAstV1 antibody at 24 hpi. From this value, the multiplicity of infection (MOI) was calculated to be approximately 0.22.

### Infection and drug treatment

Prior to infection, confluent Caco-2 cells maintained in EMEM + were washed with PBS (−) thrice and starved of serum for 1 h by incubation in EMEM supplemented with sodium pyruvate, non-essential amino acids, and 20 mM HEPES (EMEM^−^). HAstV1 stock was pretreated with 10 μg/mL trypsin IV (catalog no. T4799; Sigma-Aldrich) for 15 min at 37°C, and then applied to the cells along with trypsin at approximately 100 particles per cell. The mixture was then incubated for 1 h at 4°C, which was intended to allow the virus to bind the cells, but not proceed further in the entry process. We noted that this procedure has been described in Moser and Schulz-Cherry [19] and that incubation at 4°C for 1 h did not substantially alter the infectious events seen when incubating at 37°C, judged by the number of cells positive for viral antigen after staining with mouse anti-HAstV1 antibody (Data not shown). After removal of the culture medium and washing with EMEM^–^, incubation of the cells was continued in EMEM^–^ supplemented with 10 μg/mL trypsin IV until the time of harvest. For experiments involving pharmacological inhibitors, the infection of Caco-2 cells was carried out in the presence of a specified drug for a designated time period (i.e., with the inhibitor present during both the incubation with virus and the subsequent EMEM^–^/trypsin IV incubation).

Genistein, U0126, JNK inhibitor II, H-89, Akt inhibitor V (triciribine), and Y-27632 were purchased from Merck (Whitehouse Station, NJ, USA). Wortmannin and staurosporine were from Sigma-Aldrich. SB203580 and LY294002 were obtained from Promega (Fitchburg, WI, USA). NSC23766 and MK-2206 were from Santa Cruz Biotechnology (Santa Cruz, CA, USA) and Selleckchem (Houston, TX, USA), respectively. All drugs were solubilized in dimethyl sulfoxide (DMSO). Initial drug concentrations were selected after consulting the following references: staurosporine [41], genistein [42], U0126 [19,43], SB205830 [19], JNK inhibitor II [44], LY294003 [43], wortmannin [45], triciribine [46], MK-2206 [47], NSC23766 [48], Y-27632 [43], and H-89 [49]. The appropriate concentrations of some drugs were determined empirically by examining their inhibitory effect on HAstV1 infection using immunofluoresent detection of viral capsid-positive cells (Additional file 3) or ELISA for the extent of viral capsid proteins released from HAstV1-infected Caco-2 cells infected with HAstV1 (Additional file 2).

### Immunofluorescence detection of viral capsid protein

Infected cells were fixed with either acetone-methanol or 4% paraformaldehyde in PBS without magnesium or calcium, PBS(−), and reacted with mouse anti-HAstV IgG (catalog no. sc-53559; Santa Cruz Biotechnology) in PBS containing 0.5% TritonX-100. Goat anti-mouse IgG conjugated with AlexaFluor 488 (catalog no. A-11017; Invitrogen,) was used as the secondary antibody. Immunostained cells were examined under the epifluorescent microscope BZ1000 (Keyence, Osaka, Japan) and immunofluorescence images were prepared using Adobe Photoshop (Adobe Systems, Mountain View, CA, USA). For quantitation of viral infection, approximately two hundred cells were counted in at least three different areas, and the proportion of HAstV1 capsid-positive cells within the counted cells was used for statistical analysis (see below).

### Measurement of cell viability

Viability of cells infected with HAstV1 in the absence or presence of inhibitors was examined using a cell proliferation assay kit (WST-1 cell proliferation assay kit; Takara, Otsu, Japan), which is based on the cleavage of a tetrazolium salt by mitochondrial dehydrogenases to form formazan in viable cells. Designated dose of WST-1 [2-(4-Iodophenyl)-3-(4-nitrophenyl)-5-(2,4-disulfophenyl)-2H-tetrazolium] was added to the cell culture at 20 hpi and incubation was continued for an additional 4 h. The cell culture medium was then measured for absorbance at 450 nm versus a 650 nm reference using a SpectraMax M5 microplate reader (Molecular Devices, Sunnyvale, CA, USA).

### Western blot analysis of phosphorylated MAPKs and Akt

The protein content of infected cell lysates was quantified by either the Bradford method (Takara) using a BCA Protein Quantitation Kit (Thermo Fisher Scientific, Waltham, MA, USA) or the Qubit fluorometric quantitation system for protein (Invitrogen). Then, cell lysate samples containing the same amount of protein were separated using 12.5% SDS-polyacrylamide gels, transferred onto PVDF membranes, and probed for MAPKs or Akt using specific antibodies. The primary antibodies, all obtained from Cell Signaling (Beverly, MA, USA) include the following: three rabbit antibodies from the MAPK family antibody sampler kit (catalog no. 9926S), anti-p44/42 MAPK (ERK1/2; clone 137 F5), anti-SAPK/JNK (clone 56G8), or anti-p38 MAPK; three rabbit antibodies from the Phospho MAPK family antibody sampler kit (catalog no. 9910S), anti-phospho-p38 MAPK (Thr180/Tyr182; clone D2F9), anti-phospho-p44/42 MAPK (ERK1/2; Thr202/Tyr294; clone D13.14.4E), or anti-phospho-SAPK/JNK (Thr183/Tyr185; clone 81E11); rabbit anti-Akt antibody (catalog no. 4691; clone C67E7); and anti-phospho-Akt (Ser473) antibody (catalog no. 4060; clone D9E). A secondary antibody against rabbit IgG, conjugated with horseradish peroxidase (HRP; Cell Signaling) was used in all cases, and signal was detected using enzyme-linked chemiluminescence with Immunostar (Wako Pure Chemical Industries, Osaka, Japan) and exposing the blot to X-ray film to visualize bands. The membranes were first probed for phosphorylated kinases, and then reprobed for total amount of kinases. Restore Plus Western Blot Stripping Buffer (Thermo Fisher Scientific, Waltham, MA, USA) was used to strip the antibodies from the blot. The chemiluminescent signal was quantified from densitometric readings of digital images (using ImageJ software) retrieved by scanning the X-ray film.

### Quantitation of viral RNA present in cells and cell culture supernatants

RNA was purified from infected cells using the Nucleospin RNA Kit (Macherey-Nagel, Düren, Germany). The extracted RNA was quantified using a spectrophotometer (Nanodrop2000; Thermo Fisher Scientific), and a fixed amount of total RNA was used for quantitation of viral RNA. For culture supernatants, RNA was purified from the conditioned medium collected 24 h after infection using the QIAamp Viral RNA Mini Kit (Qiagen). The viral RNA was quantified using the OneStep SYBR PrimeScript Plus RT-PCR Kit with the primer set S3988-4008 and AS 4193–4171 (see above), along with a known amount of in vitro transcribed HAstV1 RNA as a standard. The level of amplification of the ORF1 region was then converted to the quantity of full-length viral RNA.

### Enzyme-linked immunobsorbant assay (ELISA) for viral capsid

The culture supernatants of infected cells were examined for the presence of viral capsid by ELISA. In brief, 50 μL of conditioned medium from infected cultures was applied to each well, incubated overnight at 4°C in microtiter plates (Nunc-Immuno Plate MaxiSorp; Thermo Fisher Scientific), washed with PBS (−) containing 0.1% Tween 20, and incubated with mouse anti-HAstV IgG (3 μg/ml) in a blocking solution (StartingBlock Blocking Buffer in TBS with Tween 20; catalog no. 37543; Thermo Fisher Scientific) for 1 h at 37°C. After being washed, the wells were incubated with a 5000-fold dilution of HRP-conjugated sheep anti-mouse antibody (GE Healthcare, Waukesha, WI, USA) in the blocking solution for 1 h at 37°C, followed by incubation with an HRP-colorimetric substrate (TMB Microwell Peroxidase Substrate System; catalog no. 50-76-00; KPL, Gaithersburg, MD, USA) at room temperature. The colorimetric reaction was stopped using TMB Stop Solution (catalog no. 50-85-05; KPL) and the absorbance was measured using a SpectraMax M5 microplate reader (Molecular Devices).

### Statistical analysis

ANOVA was used to examine statistical variance between experimental groups. The variance between individual set of data were examined by Student’s *t*-test. *P* values of < 0.01 or <0.05 were considered significant and indicated in graphs.

## Competing interests

The authors declare that they have no competing interests.

## Author’s contributions

ST and AN designed the experiments. AN wrote the manuscript. ST, YZ, YN, and AN conducted the experiments. TI helped prepare the experiments. All authors read and approved the final manuscript.

## Supplementary Material

Additional file 1 **Blockade of Akt phosphorylation at 24 hpi in HAstV1-infected Caco-2 cells by inhibitors of PI3K and Akt.** Caco-2 cells infected with HAstV1 were incubated for 24 h in the presence or absence of the indicated inhibitors. The cells were then harvested, and equal amount of the protein was separated through 12.5% SDS-polyacrylamide gels, followed by transfer to a PVDF membrane for Western blot. The membrane was probed for phosphorylated Akt (pAKT) and then reprobed for total Akt (Akt), as described in the Methods section. Click here for file

Additional file 2 **Effects of varying drugs doses on the extent of HAstV1 capsid release at 24 hpi.** The dose–response effects on HAstV1 infection from treatment with genistein, U0126, LY294002, wortmannin, triciribine, MK2206, H89, and Y27632 were examined by measuring viral capsid release in culture supernatants at 24 hpi using ELISA. Drug concentrations (μM) are indicated at the bottom of each bar. Each bar indicates a value relative to that obtained with treatment of solvent alone (DMSO). The mean of three different samples is shown with the standard deviation. Values indicating a statistically significant difference from “mock” are marked (*P < 0.05; **P < 0.01). Click here for file

Additional file 3 **Effects of varying drug doses on viral capsid expression 24 h after HAstV1 infection.** Kinase inhibitors, at different concentrations, were added to Caco-2 cells upon HAstV1 infection, and the effects on the viral capsid expression were examined as in Figure 1. The proportion of cells positive for viral capsid in a sample of approximately 200 cells was divided by the proportion obtained from cells infected with HAstV1 alone (“Mock”). The mean values obtained by counting at three different spots on the coverslip is shown as a bar; error bars represent the standard deviation. The drug and the concentration used is shown at the bottom of each bar. Values that show a statistically significant difference from that of the “mock” are marked as ** (P < 0.01). Click here for file
