# Supplementary figures and images for: Initiation of human astrovirus type 1 infection was blocked by inhibitors of phosphoinositide 3-kinase
Source: Virol J. 2013 May 16;10:153. doi: 10.1186/1743-422X-10-153 (PMC3750554; doi:10.1186/1743-422X-10-153)

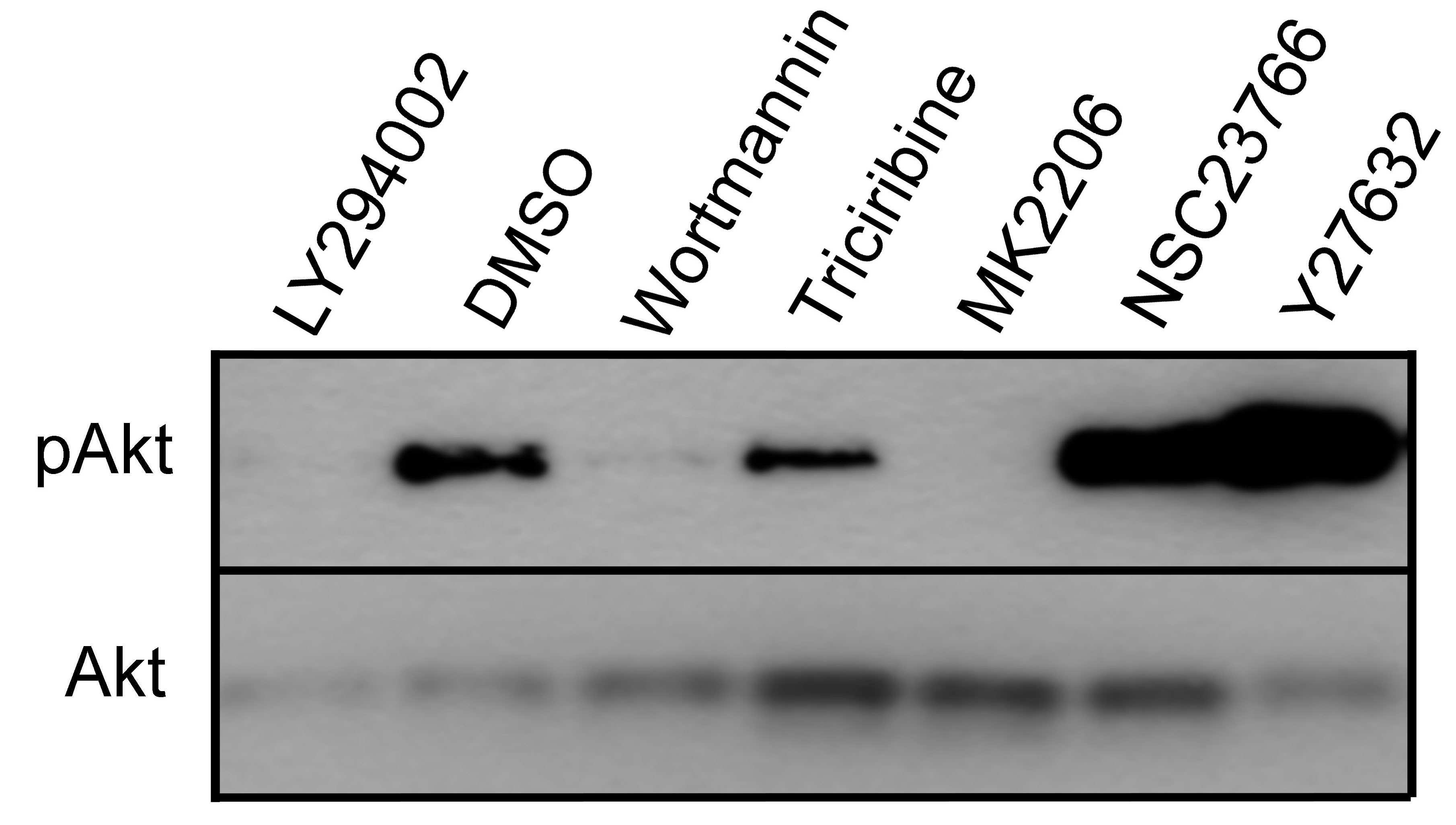

Supplement: Additional file 1 — Blockade of Akt phosphorylation at 24 hpi in HAstV1-infected Caco-2 cells by inhibitors of PI3K and Akt. Caco-2 cells infected with HAstV1 were incubated for 24 h in the presence or absence of the indicated inhibitors. The cells were then harvested, and equal amount of the protein was separated through 12.5% SDS-polyacrylamide gels, followed by transfer to a PVDF membrane for Western blot. The membrane was probed for phosphorylated Akt (pAKT) and then reprobed for total Akt (Akt), as described in the Methods section. [file 1743-422X-10-153-S1.jpeg]

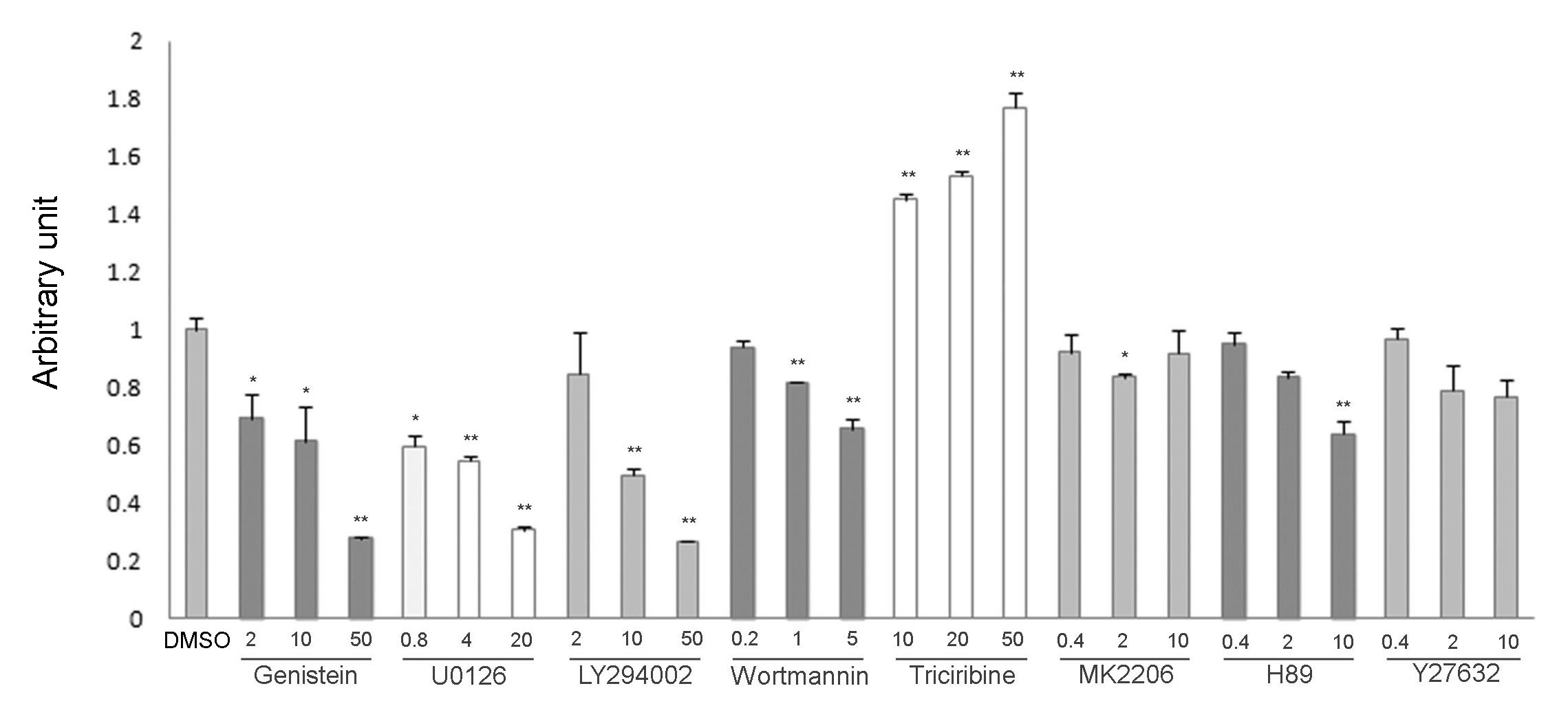

Supplement: Additional file 2 — Effects of varying drugs doses on the extent of HAstV1 capsid release at 24 hpi. The dose–response effects on HAstV1 infection from treatment with genistein, U0126, LY294002, wortmannin, triciribine, MK2206, H89, and Y27632 were examined by measuring viral capsid release in culture supernatants at 24 hpi using ELISA. Drug concentrations (μM) are indicated at the bottom of each bar. Each bar indicates a value relative to that obtained with treatment of solvent alone (DMSO). The mean of three different samples is shown with the standard deviation. Values indicating a statistically significant difference from “mock” are marked (*P < 0.05; **P < 0.01). [file 1743-422X-10-153-S2.jpeg]

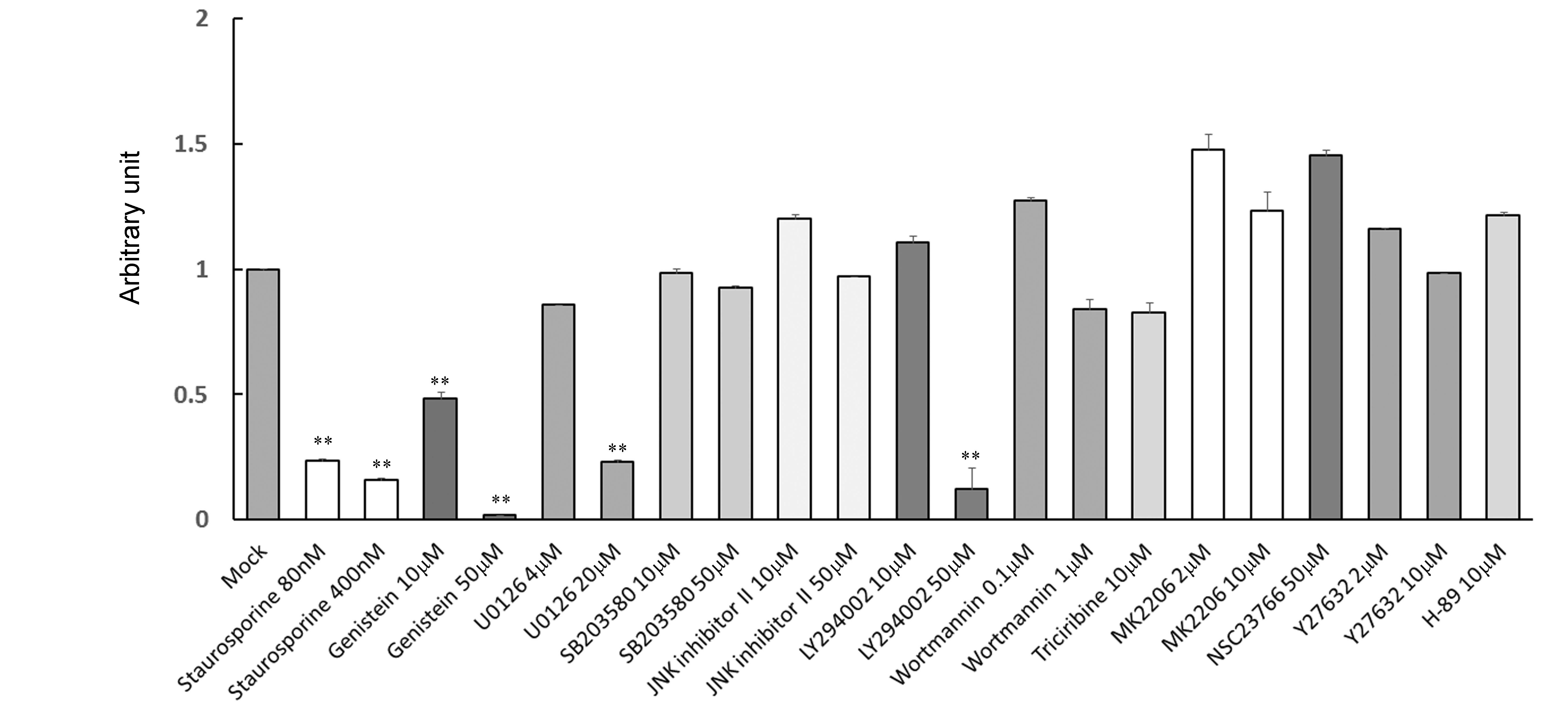

Supplement: Additional file 3 — Effects of varying drug doses on viral capsid expression 24 h after HAstV1 infection. Kinase inhibitors, at different concentrations, were added to Caco-2 cells upon HAstV1 infection, and the effects on the viral capsid expression were examined as in Figure 1. The proportion of cells positive for viral capsid in a sample of approximately 200 cells was divided by the proportion obtained from cells infected with HAstV1 alone (“Mock”). The mean values obtained by counting at three different spots on the coverslip is shown as a bar; error bars represent the standard deviation. The drug and the concentration used is shown at the bottom of each bar. Values that show a statistically significant difference from that of the “mock” are marked as ** (P < 0.01). [file 1743-422X-10-153-S3.jpeg]
